# Supplementary material for: Assessing reliability and accuracy of qPCR, dPCR and ddPCR for estimating mitochondrial DNA copy number in songbird blood and sperm cells
Source: PeerJ. 2025 Apr 11;13:e19278. doi: 10.7717/peerj.19278 (PMC11995889; doi:10.7717/peerj.19278)
Supplement: Supplemental Information 1 [file peerj-13-19278-s001.docx]

**Supplementary Information**

**Table S1.** GenBank accession numbers of the sequences used for primer design.

| **Species** | **GenBank accession numbers** |
| --- | --- |
| Hawfinch (*Coccothraustes coccothraustes*) | KX109614.1; NC_025614.1; KM078789.1 |
| Great tit (*Parus major*) | HQ417139.1; FJ465247.1; AJ224494.1; KP137624.1; NC_040875.1 |
| Common reed bunting (*Emberiza schoeniclus*) | KP877661.1; OQ274893.1; MW417350.1; NC_062087.1; MT081419.1 |
| Eurasian chaffinch (*Fringilla coelebs*) | HQ284765.1; NC_025599.1; MN122830.1; KM078769.1 |
| Eurasian bullfinch (*Pyrrhula pyrrhula*) | HQ284732.1; NC_025625.1; KM078804.1; HQ284731.1; HQ284730.1 |
| Bluethroat (*Luscinia svecica*) | MN122892.1 |
| Eurasian siskin (*Spinus spinus*) | NC_015198.1; HQ915866.1 |

**Table S2.** Sperm samples used for PCR and ddPCR validation of the primers.

| **Species** | **Accession number** | **Collection date** | **Collection location** |
| --- | --- | --- | --- |
| *Spinus spinus* | NHMO-BI-106957  NHMO-BI-108466  NHMO-BI-108463 | 28/04/2021  02/06/2022  02/06/2022 | 59.744 N \| 10.820 E  61.226 N \| 9.040 E  61.226 N \| 9.040 E |
| *Turdus pilaris* | NHMO-BI-106959 | 08/06/2021 | 61.25 N \| 9.06 E |
| *Phylloscopus trochilus* | NHMO-BI-106966 | 17/06/2021 | 61.22 N \| 9.04 E |
| *Luscinia svecica* | NHMO-BI-100507 | 24/05/2018 | 61 25 N \| 8 52 E |
| *Fringilla montifringilla* | NHMO-BI-106797  NHMO-BI-108304 | 21/05/2021  04/06/2022 | 61.226 N \| 9.040 E  61.226 N \| 9.040 E |
| *Pyrrhula pyrrhula* | NHMO-BI-104533 | 19/06/2020 | 61.226 N \| 9.040 E |
| *Regulus regulus* | NHMO-BI-108611  NHMO-BI-108615 | 07/05/2022  13/05/2022 | 59.98 N \| 10.74 E  59.99 N \| 10.79 E |
| *Coccothraustes coccothraustes* | NHMO-BI-107668  NHMO-BI-107669 | 19/05/2022  19/05/2022 | 59.744 N \| 10.820 E  59.744 N \| 10.820 E |
| *Emberiza schoeniclus* | NHMO-BI-108669  NHMO-BI-108534 | 21/05/2022  15/05/2022 | 61 25 N \| 8 52 E  61.226 N \| 9.040 E |

**Table S3.** Sperm and blood samples used in the study. Ten sperm samples from 8 individuals and 10 blood samples from 10 individuals were used in the study. Two individuals were sampled twice for sperm (NHMO-BI-108302 and NHMO-BI-108464).

| Sample type | Accession number | Species | Collection date | Collection location |
| --- | --- | --- | --- | --- |
| Sperm | NHMO-BI-108302 | *Spinus spinus* | 02/06/2022 | 61.226 N \| 9.040 E |
| Sperm | NHMO-BI-108302 | *Spinus spinus* | 02/06/2022 | 61.226 N \| 9.040 E |
| Sperm | NHMO-BI-108301 | *Spinus spinus* | 02/06/2022 | 61.226 N \| 9.040 E |
| Sperm | NHMO-BI-108479/2-S | *Spinus spinus* | 13/06/2022 | 61.226 N \| 9.040 E |
| Sperm | NHMO-BI-108464/1-S | *Spinus spinus* | 02/06/2022 | 61.226 N \| 9.040 E |
| Sperm | NHMO-BI-108464 | *Spinus spinus* | 02/06/2022 | 61.226 N \| 9.040 E |
| Sperm | NHMO-BI-106779/1-S | *Spinus spinus* | 11/06/2021 | 61.226 N \| 9.040 E |
| Sperm | NHMO-BI-106744/1-S | *Spinus spinus* | 11/05/2021 | 61.226 N \| 9.040 E |
| Sperm | NHMO-BI-106748/1-S | *Spinus spinus* | 17/05/2021 | 61.226 N \| 9.040 E |
| Sperm | NHMO-BI-106746/1-S | *Spinus spinus* | 13/05/2021 | 59.744 N \| 10.820 E |
| Blood | NHMO-BI-106927/1-B | *Spinus spinus* | 13/05/2021 | 59.744 N \| 10.820 E |
| Blood | NHMO-BI-108472/1-B | *Spinus spinus* | 13/06/2022 | 61.226 N \| 9.040 E |
| Blood | NHMO-BI-108479/1-B | *Spinus spinus* | 13/06/2022 | 61.226 N \| 9.040 E |
| Blood | NHMO-BI-108493/1-B | *Spinus spinus* | 22/06/2022 | 61.226 N \| 9.040 E |
| Blood | NHMO-BI-109025/1-B | *Spinus spinus* | 13/07/2022 | 61.226 N \| 9.040 E |
| Blood | NHMO-BI-109030/1-B | *Spinus spinus* | 13/07/2022 | 61.226 N \| 9.040 E |
| Blood | NHMO-BI-109034/1-B | *Spinus spinus* | 13/07/2022 | 61.226 N \| 9.040 E |
| Blood | NHMO-BI-109036/1-B | *Spinus spinus* | 13/07/2022 | 61.226 N \| 9.040 E |
| Blood | NHMO-BI-109039/1-B | *Spinus spinus* | 13/07/2022 | 61.226 N \| 9.040 E |
| Blood | NHMO-BI-109040/1-B | *Spinus spinus* | 13/07/2022 | 61.226 N \| 9.040 E |

**Table S4.** The LOD and LOQ values for synthetic DNA concentrations with the three different PCR methods. The method outlined in [1] was used to assess LOD and LOQ for qPCR. The LOD for ddPCR, dPCR and qPCR has been calculated using the script provided in the supplementary material as in [2]. Both methods show  similar LOD values for qPCR: 2.4 copies/µL using the method described in [1] and 2.417 copies/µL using the method described by Hunter et al. [2]. To establish  LOQs for dPCR or ddPCR, we followed the method outlined in [3] and [4], and considered LOQ as the lowest concentration at which the CV is ≤ 35%.

| **Method** | **LOD (copies/µL)** |  | **LOQ (copies/ µL)** |
| --- | --- | --- | --- |
| dPCR | 0.51 |  | 2.12 |
| ddPCR | 0.63 |  | 1.23 |
| qPCR | 2.42 |  | 4.9 |

**Table S5.** Calculated copy number of synthetic mtDNA. Based on the molecular weight (30854 g/mole) of the synthetic oligo, the number of copies (molecules) for the different dilutions of the synthetic DNA was estimated using the formula provided below the table.

| **Dilution steps (ng/µL)** | **Copy number (copies/µL)** |
| --- | --- |
| 0.00025 | 4879513 |
| 0.000025 | 487951.3 |
| 2.5E-06 | 48795.13 |
| 2.5E-07 | 4879.513 |
| 2.5E-08 | 487.9513 |
| 2.5E-09 | 48.79513 |
| 2.5E-10 | 4.879513 |
| 2.5E-11 | 0.487951 |
| 2.5E-12 | 0.048795 |


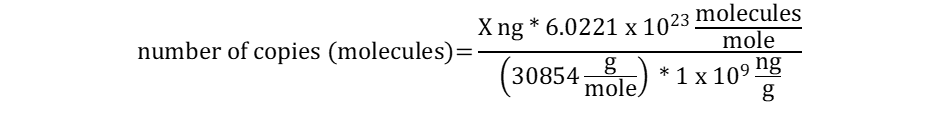


**
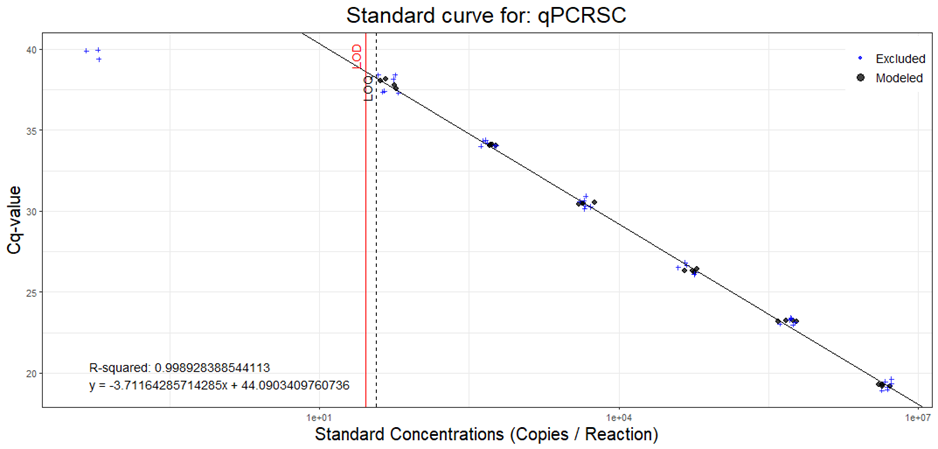
**

**Figure S1.** Estimated Limit of Detection (LOD) and Limit of Quantification (LOQ) for qPCR. The calculations and the figure were generated following the R script provided in [1].

**
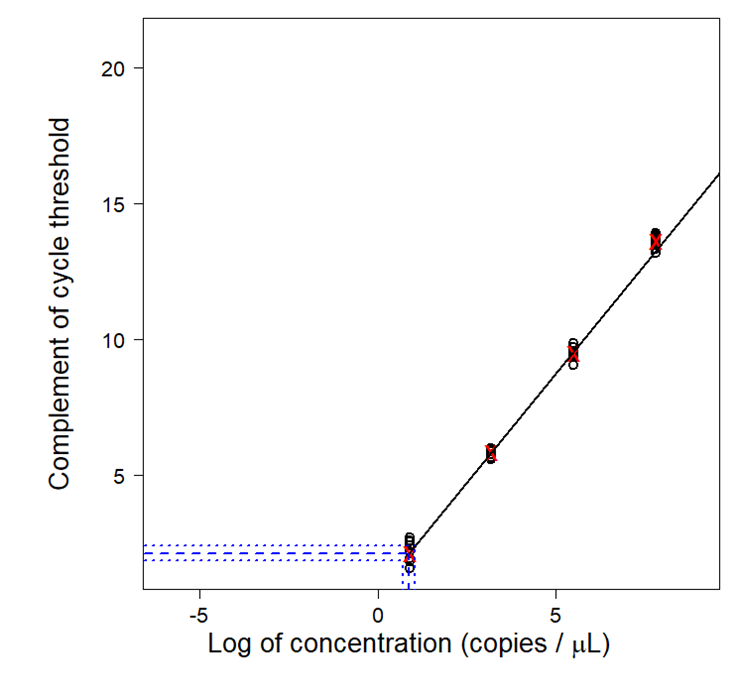
**

**Figure S2.** Limit of Detection estimation for qPCR. Figure and calculations were performed  following the method outlined in [2].


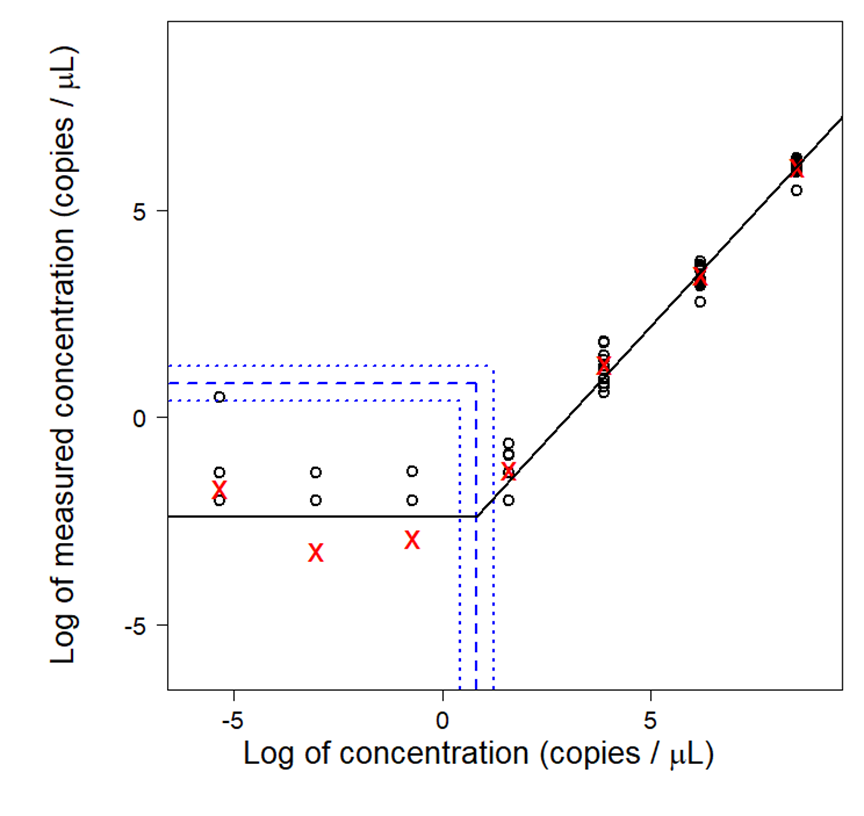


**Figure S3.** Limit of detection estimation for dPCR. Figure and calculations were performed following the method outlined in [2].


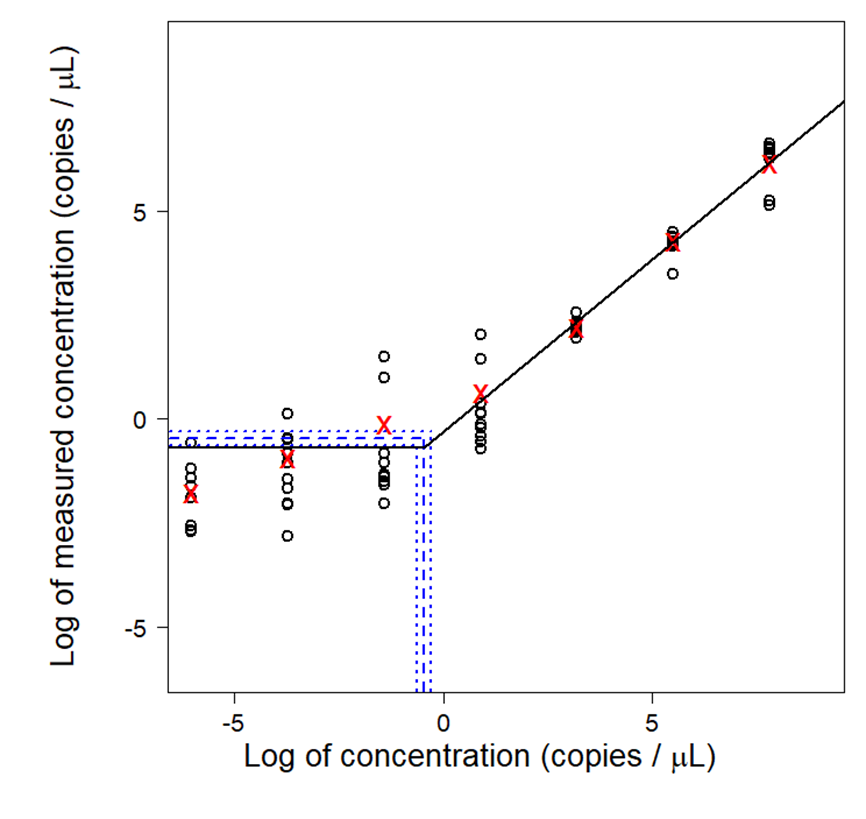


**Figure S4.** Limit of Detection estimation for ddPCR. Figure and calculations were performed following the method outlined in [2].


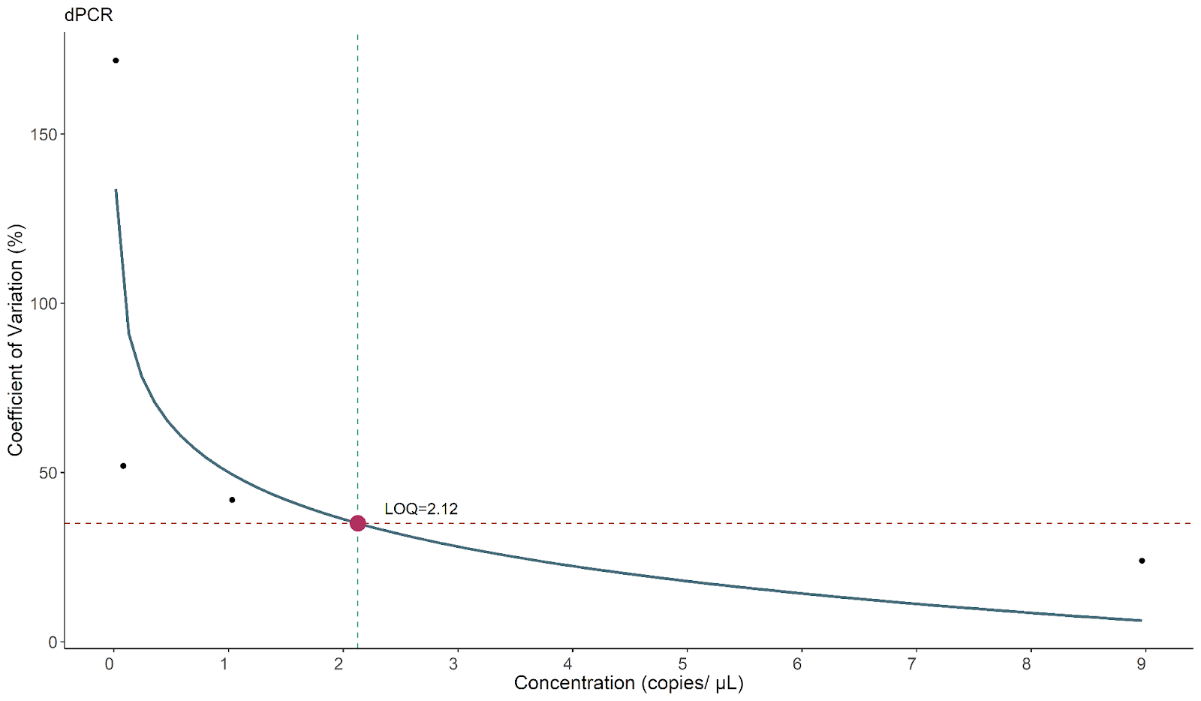


**Figure S5.** Limit of Quantification (LOQ) of dPCR.


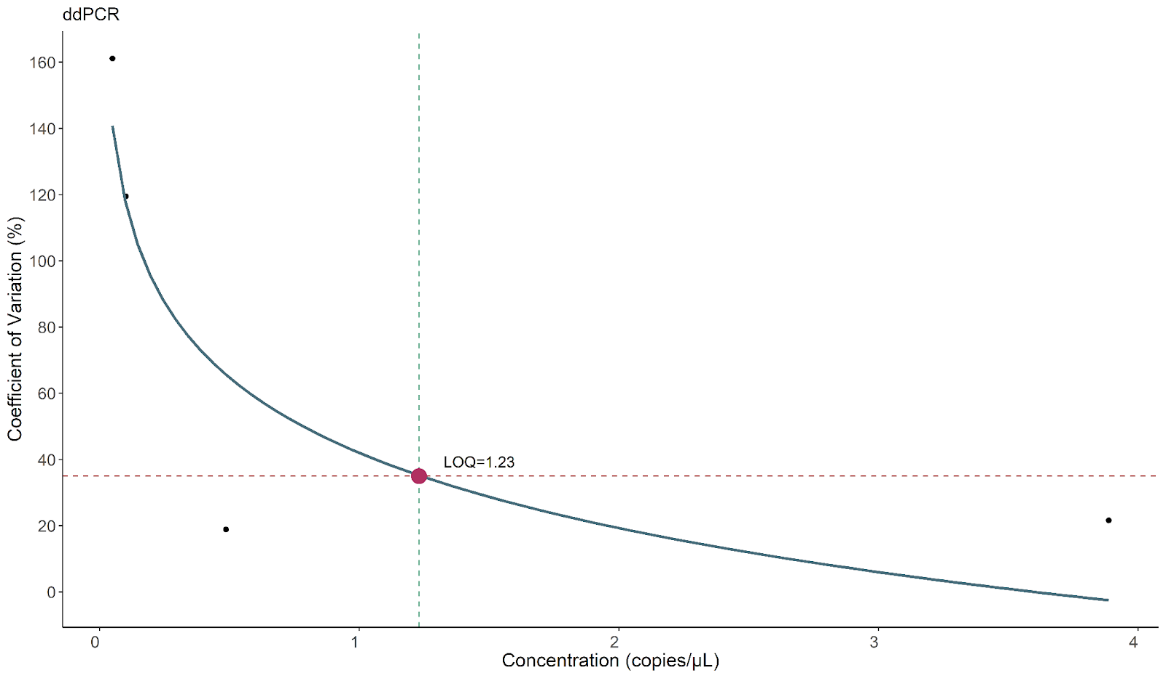


**Figure S6.** Limit of Quantification (LOQ) of ddPCR.

**References**

1. Klymus KE, Merkes CM, Allison MJ, Goldberg CS, Helbing CC, Hunter ME, Jackson CA, Lance RF, Mangan AM, Monroe EM, Piaggio AJ, Stokdyk JP, Wilson CC, Richter CA (2020) Reporting the limits of detection and quantification for environmental DNA assays. Environ DNA 2:271–282. https://doi.org/10.1002/edn3.29

2. Hunter ME, Dorazio RM, Butterfield JSS, Meigs-Friend G, Nico LG, Ferrante JA (2017) Detection limits of quantitative and digital PCR assays and their influence in presence–absence surveys of environmental DNA. Mol Ecol Resour 17:221–229. https://doi.org/10.1111/1755-0998.12619

3. Forootan A, Sjöback R, Björkman J, Sjögreen B, Linz L, Kubista M (2017) Methods to determine limit of detection and limit of quantification in quantitative real-time PCR (qPCR). Biomol Detect Quantif 12:1–6. https://doi.org/10.1016/j.bdq.2017.04.001

4. Brys R, Halfmaerten D, Neyrinck S, Mauvisseau Q, Auwerx J, Sweet M, Mergeay J (2021) Reliable eDNA detection and quantification of the European weather loach (Misgurnus fossilis). J Fish Biol 98:399–414. https://doi.org/10.1111/jfb.14315
