# Supplementary material for: Assessing reliability and accuracy of qPCR, dPCR and ddPCR for estimating mitochondrial DNA copy number in songbird blood and sperm cells
Source: PeerJ. 2025 Apr 11;13:e19278. doi: 10.7717/peerj.19278 (PMC11995889; doi:10.7717/peerj.19278)
Supplement: Supplemental Information 3 [file peerj-13-19278-s003.docx]

#**Figure 2** The relationship between log-transformed expected copy number of the synthetic oligo and log-transformed copy numbers obtained with dPCR and ddPCR.

siskin_palette <- c("#EBD828", "#9F9A46", "#81746F") ## newesr palette

synthetic<- read.csv("Data_sheet_all_info_Synthetic_and_Real/Synthetic_DNA-ALL.csv") #

Subs_Synth <- synthetic[synthetic$percent_positive < 95 & (synthetic$Method == "dPCR" | synthetic$Method == "ddPCR"), ]

filter_subs_synth <- Subs_Synth[Subs_Synth$Concentration.ng.uL != "2.50E-05", ]

Concentrations4 <- ggplot(filter(filter_subs_synth, !Concentration.ng.uL %in% c("NTC")),

aes(x = log(expected_concentration), y = log(Concentration_Cq), color = Method)) +

geom_point(size = 2) +

geom_smooth(method = "lm", formula = y ~ poly(x, 2), se = FALSE) + # Polynomial regression line

theme_light() +

theme_classic() +

theme(axis.text.x = element_text(angle = 0, vjust = 0.5, hjust = 1, size = 12),

axis.text.y = element_text(size = 12),

axis.title = element_text(size = 14),

legend.text = element_text(size = 12)) +

geom_abline(intercept = 0, slope = 1, linetype = "dotted", color = "black", linewidth = 1) +

labs(x = "Log Expected Copy Number (copies/µL)", y = "Log Measured Copy Number (copies/µL)")+

#geom_abline(intercept = 0, slope = 1, linetype = "dotted", color = "black",linewidth=1) +

#scale_y_continuous(limits = c(0,10)) +

scale_color_manual(values = siskin_palette) +

# guides(color = "none") +

theme_bw(base_size = 20)+

guides(color = "none") # remove the legend for color

Concentrations4

#**Figure 3** Comparison of qPCR, dPCR and ddPCR quantification of mtDNA copy number in sperm and blood samples.

Real_data_<-read.csv("02_real_DNA_methods-transformed.csv")

# to plot in the order I want, I can convert my method variable into factors and choose the desired order

Real_data_$Method <- factor(Real_data_$Method, levels = c("qPCR", "dPCR", "ddPCR"))

Sperm_concentration <- ggplot(subset(Real_data_, DNA_type %in% "Sperm "), aes(x = Method, y = Mean_conc, fill= Method))+

theme_light()+

theme_classic()+

geom_boxplot(fill = siskin_palette)+

labs(y= "Mean concentration (copies/µL)", x="")+

ggtitle("Sperm")+

theme(axis.text.x = element_text(angle = 90, vjust = 0.5, hjust = 1, size = 12), # Adjust the size as needed

axis.text.y = element_text(size = 12), # Adjust the size as needed

axis.title = element_text(size = 14), # Adjust the size as needed

legend.position = "none") +

theme_bw(base_size = 20)

Sperm_concentration

#mean concentration for blood

Blood_concentration <- ggplot(subset(Real_data_, DNA_type %in% "Blood"), aes(x = Method, y = Mean_conc, fill= Method))+

theme_light()+

theme_classic()+

geom_boxplot(fill = siskin_palette)+

# theme(axis.text.x = element_text(angle = 90, vjust = 0.5, hjust=1))+

labs(y= "", x="")+

ggtitle("Blood") +

# scale_y_continuous(limits = c(0,100), breaks = seq(0,100, 50))+

theme(legend.position = "none") +

theme(axis.text.x = element_text(angle = 90, vjust = 0.5, hjust = 1, size = 12), # Adjust the size as needed

axis.text.y = element_text(size = 12), # Adjust the size as needed

axis.title = element_text(size = 14), # Adjust the size as needed

legend.position = "none") +

theme_bw(base_size = 20)

Blood_concentration

plot_grid(Sperm_concentration,Blood_concentration)

# **Figure 4** Comparison of coefficient of variation in mtDNA copy number estimation in sperm and blood across samples, every sample was quantified in triplicate. qPCR (black diamond), dPCR (olive green triangle) and ddPCR (yellow circle).

library(ggplot2)

library(dplyr)

library(cowplot)

data <- read.csv("02_real_DNA_methods-transformed.csv")

sperm_data <- filtered_data %>%

filter(DNA_type == "Sperm ")

sperm_data$individual <- factor(sperm_data$individual) ##To make the x-axis reflect the 10 individuals in your data, you need to specify individual as a factor variable.

CV_sperm<- ggplot(sperm_data, aes(x = individual, y = CV_., color = Method, shape = Method)) +

geom_point(size = 4) +

labs(x = "Individual", y = "Coefficient of Variation (%)", color = "Method", shape = "DNA type") +

scale_color_manual(values = c("dPCR" = "#9F9A46", "qPCR" = "#141412", "ddPCR" = "#EBD828")) +

scale_shape_manual(values = c(16, 17, 18)) +

theme_minimal() +

scale_y_continuous(limits = c(0, 100)) + # Set y-axis limits

theme(axis.text.x = element_text(angle = 45, vjust = 1, hjust = 1, size = 12),

axis.text.y = element_text(size = 12),

axis.title = element_text(size = 14),

legend.text = element_text(size = 13)) +

theme_bw(base_size = 20) +

guides(color = FALSE, shape = FALSE)+

ggtitle("Sperm")

CV_sperm

### bloood

blood_data <- filtered_data %>%

filter(DNA_type == "Blood")

blood_data$individual <- factor(blood_data$individual) ##To make the x-axis reflect the 10 individuals in your data, you need to specify individual as a factor variable.

CV_blood<- ggplot(blood_data, aes(x = individual, y = CV_., color = Method, shape = Method)) +

geom_point(size = 4) +

labs(x = "Individual", y = "", color = "Method", shape = "DNA type") +

scale_color_manual(values = c("dPCR" = "#9F9A46", "qPCR" = "#141412", "ddPCR" = "#EBD828")) +

scale_shape_manual(values = c(16, 17, 18)) +

theme_minimal() +

scale_y_continuous(limits = c(0, 100)) + # Set y-axis limits

theme(axis.text.x = element_text(angle = 45, vjust = 1, hjust = 1, size = 12),

axis.text.y = element_text(size = 12),

axis.title = element_text(size = 14),

legend.text = element_text(size = 13)) +

theme_bw(base_size = 20) +

guides(color = FALSE, shape = FALSE)+

ggtitle("Blood")

CV_blood

plot_grid(CV_sperm, CV_blood)

#**Figure 5** The relationship between coefficient of variation (CV) and log-transformed mean mtDNA concentration measurement of blood and sperm samples across the three quantification platforms.

#siskin_palette <- c("#EBD828", "#9F9A46", "#81746F")

data <- read.csv("02_real_DNA_methods-transformed.csv")

CV_conc <- ggplot(filtered_data, aes(x = log(Mean_conc), y = CV_., color = Method, shape = DNA_type)) +

geom_point(size = 4) +

geom_smooth(method = "lm", se = FALSE, aes(group = Method), linetype = "dashed") + # Add regression lines for each Method

labs(x = " Log Concentration (copies/µL)", y = "Coefficient of Variation (%)", color = "Method", shape = "DNA type") +

scale_color_manual(values = c("dPCR" = "#9F9A46", "qPCR" = "#141412", "ddPCR" = "#EBD828")) + # Assign colors to each Method

scale_shape_manual(values = c(16, 17, 18)) + # Apply different shapes for DNA types

theme_minimal() +

theme(axis.text.x = element_text(angle = 0, vjust = 0.5, hjust = 1, size = 12), # Adjust the size as needed

axis.text.y = element_text(size = 12), # Adjust the size as needed

axis.title = element_text(size = 14),

legend.text = element_text(size = 13)) +

theme_bw(base_size = 20)+#

guides(color = FALSE, shape = FALSE) # Remove both color and shape legends

CV_conc

## Figures presented in Supplementary information: Figure S1 – S6

#**Figure S1** Estimated Limit of Detection (LOD) and Limit of Quantification (LOQ) for qPCR. The calculations and the figure were generated following the R script provided in Klymus KE, Merkes CM, Allison MJ, Goldberg CS, Helbing CC, Hunter ME, Jackson CA, Lance RF, Mangan AM, Monroe EM, Piaggio AJ, Stokdyk JP, Wilson CC, Richter CA (2020) Reporting the limits of detection and quantification for environmental DNA assays. Environ DNA 2:271–282. https://doi.org/10.1002/edn3.29

## Load packages:

library(ggplot2)

library(drc)

## Set your working directory to where your csv file is saved (MODIFY AS NEEDED):

setwd("../RcodeForSupplement")

## Read in your data file (MODIFY FILE NAME AS NEEDED):

#data formatted to follow Klymus et al methods and script

DAT <- read.csv("DataLBqPCR-LOQ-Klymus.csv")

## Define your CV threshold for LoQ:

LOQ.Threshold <- 0.35

## Define which logarithmic function to use for LoD model:

LOD.FCT <- "Best"

## Selecting "Best" will signal the code to automatically select the best fitting

## model choice. Run the function getMeanFunctions() to print the list of all choices.

## Example: LOD.FCT <- W2.4()

## This example will use the Weibull type II, 4 parameter function.

## Define which model to use for LoQ model:

LOQ.FCT <- "Best"

## Selecting "Best" will signal the code to automatically select the model with lowest

## residual standard error. Change to "Decay" to use exponential decay model, "Linear"

## to use linear model, "Pn" to use an nth-order polynomial model where n is numerical.

## Example: "P2" will use a 2nd order polynomial model, "P3" will use 3rd order, and etc.

## Selecting "Best" will test polynomial models up to 6th-order.

## Create an analysis log file:

write(paste0("Analysis started: ",date(),"\n\n"),file="Analysis Log.txt")

## Check the data:

if(sum(colnames(DAT)=="Target")!=1) { #Is there a "Target" column?

A <- grep("target",colnames(DAT),ignore.case=TRUE)

if(length(A)==1) { colnames(DAT)[A] <- "Target" } #Rename target column if it is mispelled but can be identified and there is only 1.

if(length(A)!=1) { write("There is a problem with the 'Target' column.\n\n",file="Analysis Log.txt",append=TRUE) } #Add error message to analysis log.

if(length(A)>1) { cat("ERROR: multiple 'Target' columns detected.",colnames(DAT)[A],sep="\n") }

if(length(A)==0) { print("ERROR: cannot detect 'Target' column.") }

}

if(sum(colnames(DAT)=="Cq")!=1) { #Is there a "Cq" column?

A <- grep("cq|ct|cycle",colnames(DAT),ignore.case=TRUE)

if(length(A)==1) { colnames(DAT)[A] <- "Cq" } #Rename cq column if it is mispelled but can be identified and there is only 1.

if(length(A)!=1) { write("There is a problem with the 'Cq' column.\n\n",file="Analysis Log.txt",append=TRUE) } #Add error message to analysis log.

if(length(A)>1) { cat("ERROR: multiple 'Cq' columns detected.",colnames(DAT)[A],sep="\n") }

if(length(A)==0) { print("ERROR: cannot detect 'Cq' column.") }

}

if(sum(colnames(DAT)=="SQ")!=1) { #Is there a "SQ" column?

A <- grep("sq|copies|starting|quantity",colnames(DAT),ignore.case=TRUE)

if(length(A)==1) { colnames(DAT)[A] <- "SQ" } #Rename SQ column if it is mispelled but can be identified and there is only 1.

if(length(A)!=1) { write("There is a problem with the 'SQ' column.\n\n",file="Analysis Log.txt",append=TRUE) } #Add error message to analysis log.

if(length(A)>1) { cat("ERROR: multiple 'SQ' columns detected.",colnames(DAT)[A],sep="\n") }

if(length(A)==0) { print("ERROR: cannot detect 'SQ' column.") }

}

## Ensure data is in the proper format:

DAT$Target <- as.factor(DAT$Target)

DAT$Cq <- suppressWarnings(as.numeric(as.character(DAT$Cq))) #Non-numerical values (i.e. negative wells) will be converted to NAs

DAT$SQ <- suppressWarnings(as.numeric(as.character(DAT$SQ))) #Non-numerical values (i.e. NTC) will be converted to NAs

if(sum(is.na(DAT$SQ))>0) {

write(paste0("WARNING: ",sum(is.na(DAT$SQ))," data points excluded without a valid starting quantity (SQ)!\nHere is a sample of the data being excluded:\n"),

file="Analysis Log.txt",append=TRUE)

suppressWarnings(write.table(head(DAT[is.na(DAT$SQ),]),file="Analysis Log.txt",append=TRUE,

sep="\t",eol="\n",row.names=FALSE,col.names=TRUE))

write("\n",file="Analysis Log.txt",append=TRUE)

print(paste0("WARNING: ",sum(is.na(DAT$SQ))," data points excluded without a valid starting quantity (SQ)!"))

print(head(DAT[is.na(DAT$SQ),]))

}

## Check for wild outliers that the user should go back and review:

Targets <- unique(DAT$Target)

## Get matchups of all standards and markers used:

for(i in 1:length(Targets)) {

if(i==1) {

Standards <- unique(DAT$SQ[DAT$Target==Targets[i]&!is.na(DAT$SQ)])

Target <- rep(as.character(Targets[i]),length(Standards))

}

else {

Standards <- c(Standards,unique(DAT$SQ[DAT$Target==Targets[i]&!is.na(DAT$SQ)]))

Target <- c(Target,rep(as.character(Targets[i]),

length(unique(DAT$SQ[DAT$Target==Targets[i]&!is.na(DAT$SQ)]))))

}

}

OUTS <- data.frame(Target=Target,Standard=Standards,Outliers=NA)

## Identify any wells where the Cq value is more than 10% away from the median for

## that standard.

for(i in 1:nrow(OUTS)) {

MED <- median(DAT$Cq[DAT$SQ==OUTS$Standard[i]&DAT$Target==OUTS$Target[i]],na.rm=TRUE)

A <- which(DAT$SQ==OUTS$Standard[i]&DAT$Target==OUTS$Target[i]&DAT$Cq<0.9*MED&!is.na(DAT$Cq))

B <- which(DAT$SQ==OUTS$Standard[i]&DAT$Target==OUTS$Target[i]&DAT$Cq>1.1*MED&!is.na(DAT$Cq))

if(length(c(A,B))>0) {

OUTS$Outliers[i] <- paste(c(A,B),collapse=",")

}

}

## If any outliers are detected, export the raw data as csv and make a note in

## the analysis log.

if(sum(!is.na(OUTS$Outliers))>0) {

OUT.ROW <- paste(OUTS$Outliers[!is.na(OUTS$Outliers)],collapse=",")

OUT.ROW2 <- unlist(strsplit(OUT.ROW,split=","))

write.csv(DAT[OUT.ROW2,],file="Potential-Outliers.csv",row.names=FALSE)

write("Potential outliers have been detected. Please review the data exported as

Potential-Outliers.csv, and determine if any data points need to be excluded

or adjusted due to false positives or poorly normalized baselines.",

file="Analysis Log.txt",append=TRUE)

write("\n",file="Analysis Log.txt",append=TRUE)

}

## Generate standard curves using all data and calculate copy estimates for each

## replicate using the curves:

curve.list <- ""

DAT$Copy.Estimate <- rep(NA,nrow(DAT))

DAT$Mod <- rep(0,nrow(DAT))

for(i in 1:length(Targets)) {

STDS <- data.frame(S=unique(DAT$SQ[DAT$Target==Targets[i]]),R=NA)

## Calculate detection rates for each standard:

for(j in 1:nrow(STDS)) {

STDS$R[j] <- sum(!is.na(DAT$Cq)&DAT$SQ==STDS$S[j]&DAT$Target==Targets[i],na.rm=TRUE)/sum(DAT$SQ==STDS$S[j]&DAT$Target==Targets[i],na.rm=TRUE)

}

## Only use standards with 50% or greater detection rates for linear regression:

if(sum(STDS$R>=0.5,na.rm=TRUE)>2) {

STDS2 <- STDS$S[STDS$R>=0.5&!is.na(STDS$R)&!is.na(STDS$S)]

}

## If there are not at least 3 standards with 50% or greater detection, use the top 3:

if(sum(STDS$R>=0.5,na.rm=TRUE)<3) {

STDS2 <- STDS$S[order(STDS$R,decreasing=TRUE)][1:3]

}

## Identify the 2nd and 3rd quartiles of each used standard for inclusion in the

## standard curve calculations

for(j in 1:length(STDS2)) {

D <- DAT$Cq[DAT$Target==Targets[i]&DAT$SQ==STDS2[j]]

DAT$Mod[DAT$Target==Targets[i]&DAT$SQ==STDS2[j]&DAT$Cq>=quantile(D,na.rm=TRUE)[2]&DAT$Cq<=quantile(D,na.rm=TRUE)[4]&!is.na(DAT$SQ)] <- 1

}

if(length(unique(DAT$SQ[DAT$Target==Targets[i]]))!=length(STDS2)) {

ToWrite <- paste0("These standards not included in ",Targets[i],

" standard curve regression for copy estimate calculations, because they detected below 50%: ",

paste(setdiff(unique(DAT$SQ[DAT$Target==Targets[i]]),STDS2),collapse=", "),"\n\n")

write(ToWrite,file="Analysis Log.txt",append=TRUE)

}

assign(paste0("curve",i),lm(Cq~log10(SQ),data=DAT[DAT$Target==Targets[i]&DAT$Mod==1,]))

curve.list <- c(curve.list,paste0("curve",i))

Intercept <- coef(get(curve.list[i+1]))[1]

Slope <- coef(get(curve.list[i+1]))[2]

DAT$Copy.Estimate[DAT$Target==Targets[i]] <- 10^((DAT$Cq[DAT$Target==Targets[i]]-Intercept)/Slope)

}

## Summarize the data:

DAT2 <- data.frame(Standards=Standards,Target=Target,Reps=NA,Detects=NA,Cq.mean=NA,

Cq.sd=NA,Copy.CV=NA,Cq.CV=NA)

## Fill in replicate counts, positive detect counts, mean Cq values, standard

## deviations of Cq values, and coefficient of variation of copy estimates for

## each standard and marker combination:

for(i in 1:nrow(DAT2)) {

DAT2$Reps[i] <- sum(DAT$SQ==DAT2$Standards[i]&DAT$Target==DAT2$Target[i],na.rm=TRUE)

DAT2$Detects[i] <- sum(!is.na(DAT$Cq)&DAT$SQ==DAT2$Standards[i]&DAT$Target==DAT2$Target[i],na.rm=TRUE)

DAT2$Cq.mean[i] <- mean(DAT$Cq[DAT$SQ==DAT2$Standards[i]&DAT$Target==DAT2$Target[i]],na.rm=TRUE)

DAT2$Cq.sd[i] <- sd(DAT$Cq[DAT$SQ==DAT2$Standards[i]&DAT$Target==DAT2$Target[i]],na.rm=TRUE)

DAT2$Copy.CV[i] <- sd(DAT$Copy.Estimate[DAT$SQ==DAT2$Standards[i]&DAT$Target==DAT2$Target[i]],na.rm=TRUE)/mean(DAT$Copy.Estimate[DAT$SQ==DAT2$Standards[i]&DAT$Target==DAT2$Target[i]],na.rm=TRUE)

DAT2$Cq.CV[i] <- sqrt(2^(DAT2$Cq.sd[i]^2*log(2))-1)

}

## Calculate positive detection rate for each standard and marker combination:

DAT2$Rate <- DAT2$Detects/DAT2$Reps

write("Data Summary:",file="Analysis Log.txt",append=TRUE)

suppressWarnings(write.table(DAT2,file="Analysis Log.txt",append=TRUE,sep="\t",eol="\n",

row.names=FALSE,col.names=TRUE))

## Determine the lowest standard with 95% or greater detection:

for(i in 1:length(Targets)) {

A <- min(DAT2$Standards[DAT2$Rate>=0.95&DAT2$Target==Targets[i]])

ToWrite <- paste0("For ",Targets[i],", the lowest standard with 95% or greater detection is: ",A," copies/reaction.")

ToWrite2 <- ""

if(length(which(DAT2$Rate<0.95&DAT2$Target==Targets[i]))>0) {

B <- max(DAT2$Standards[DAT2$Rate<0.95&DAT2$Target==Targets[i]])

if(B>A) {

ToWrite2 <- paste0("WARNING: For ",Targets[i],", ",B," copies/reaction standard detected at lower rate than ",A," copies/reaction standard.\nPlease retest.")

}

}

if(length(which(DAT2$Rate<0.95&DAT2$Target==Targets[i]))==0) {

ToWrite2 <- paste0("WARNING: LoD cannot be determined for ",Targets[i],", because it is lower than the lowest standard you tested.\nReport as <",A," copies/reaction, or retest with lower concentrations.")

}

write(paste0("\n\n",ToWrite,"\n"),file="Analysis Log.txt",append=TRUE)

if(ToWrite2!="") { write(paste0(ToWrite2,"\n\n"),file="Analysis Log.txt",append=TRUE) }

cat(ToWrite,ToWrite2,sep="\n")

}

## Determine LoD and LoQ by modeling, and summarize each assay:

## NOTE: LoD is now determined by dose-response modeling. Probit modeling code remains,

## but has been converted to comments.

DAT$Detect <- as.numeric(!is.na(DAT$Cq))

#LOD.list <- ""

LOD.list2 <- ""

LOD.list3 <- ""

LOQ.list <- ""

DAT3 <- data.frame(Assay=Targets,R.squared=NA,Slope=NA,Intercept=NA,Low.95=NA,

LOD=NA,LOQ=NA,rep2.LOD=NA,rep3.LOD=NA,rep4.LOD=NA,rep5.LOD=NA,rep8.LOD=NA)

LOD.FCTS <- list(LL.2(),LL.3(),LL.3u(),LL.4(),LL.5(),W1.2(),W1.3(),W1.4(),W2.2(),W2.3(),

W2.4(),AR.2(),AR.3(),MM.2(),MM.3())

for(i in 1:length(Targets)) {

## Check input suitability for probit or dose-response modeling:

if(sum(DAT2$Rate[DAT2$Target==Targets[i]]!=1&DAT2$Rate[DAT2$Target==Targets[i]]!=0)==0) {

ToWrite <- paste0("WARNING: For ",Targets[i],", all standards detected fully or failed fully. Therefore, the LoD model will not converge.")

write(paste0(ToWrite,"\n\n"),file="Analysis Log.txt",append=TRUE)

print(ToWrite)

}

if(sum(DAT2$Rate[DAT2$Target==Targets[i]]!=1&DAT2$Rate[DAT2$Target==Targets[i]]!=0)==1) {

ToWrite <- paste0("WARNING: For ",Targets[i],", only 1 standard detected in the informative range (not 0% and not 100%). Therefore, the LoD model results will be less reliable.")

write(paste0(ToWrite,"\n\n"),file="Analysis Log.txt",append=TRUE)

print(ToWrite)

}

## Define probit model:

#assign(paste0("LOD.mod",i),glm(Detect~SQ,data=DAT[DAT$Target==Targets[i],],

# family=binomial(link="probit")))

#LOD.list <- c(LOD.list,paste0("LOD.mod",i))

## Define LOQ model using lowest residual standard error selection:

if(LOQ.FCT=="Best") {

## Remove previous marker LOQ models from environment if they exist:

suppressWarnings(rm(LOQ1,LOQ2,LOQ3,LOQ4,LOQ5,LOQ6,LOQ7))

tryCatch({ #skip if model cannot be determined.

LOQ1 <- nls(Cq.CV~SSasymp(log10(Standards),Asym,R0,lrc),

data=DAT2[DAT2$Target==Targets[i],])

}, error=function(e) {

e

cat("ERROR: decay LOQ model cannot be defined for ",as.character(Targets[i]),sep="")

})

tryCatch({ #skip if model cannot be determined.

LOQ2 <- lm(Cq.CV~log10(Standards),data=DAT2[DAT2$Target==Targets[i],])

}, error=function(e) {

e

cat("ERROR: linear LOQ model cannot be defined for ",as.character(Targets[i]),sep="")

})

tryCatch({ #skip if model cannot be determined.

LOQ3 <- lm(Cq.CV~poly(log10(Standards),2),data=DAT2[DAT2$Target==Targets[i],])

}, error=function(e) {

e

cat("ERROR: 2nd polynomial LOQ model cannot be defined for ",as.character(Targets[i]),sep="")

})

tryCatch({ #skip if model cannot be determined.

LOQ4 <- lm(Cq.CV~poly(log10(Standards),3),data=DAT2[DAT2$Target==Targets[i],])

}, error=function(e) {

e

cat("ERROR: 3rd polynomial LOQ model cannot be defined for ",as.character(Targets[i]),sep="")

})

tryCatch({ #skip if model cannot be determined.

LOQ5 <- lm(Cq.CV~poly(log10(Standards),4),data=DAT2[DAT2$Target==Targets[i],])

}, error=function(e) {

e

cat("ERROR: 4th polynomial LOQ model cannot be defined for ",as.character(Targets[i]),sep="")

})

tryCatch({ #skip if model cannot be determined.

LOQ6 <- lm(Cq.CV~poly(log10(Standards),5),data=DAT2[DAT2$Target==Targets[i],])

}, error=function(e) {

e

cat("ERROR: 5th polynomial LOQ model cannot be defined for ",as.character(Targets[i]),sep="")

})

tryCatch({ #skip if model cannot be determined.

LOQ7 <- lm(Cq.CV~poly(log10(Standards),6),data=DAT2[DAT2$Target==Targets[i],])

}, error=function(e) {

e

cat("ERROR: 6th polynomial LOQ model cannot be defined for ",as.character(Targets[i]),sep="")

})

## Determine which models were able to be determined:

A <- sapply(c("LOQ1","LOQ2","LOQ3","LOQ4","LOQ5","LOQ6","LOQ7"),exists)

B <- names(A)[A==TRUE]

## If at least 1 LOQ model was determined, select the one with the lowest

## residual standard error:

if(length(B)>0) {

LOQ.res <- rep(NA,length(B))

for(j in 1:length(B)) {

LOQ.res[j] <- summary(get(B[j]))$sigma

}

C <- which(LOQ.res==min(LOQ.res,na.rm=TRUE))

assign(paste0("LOQ.mod",i),get(B[C]))

LOQ.list <- c(LOQ.list,paste0("LOQ.mod",i))

}

}

## Define LOQ model by exponential decay modeling:

if(LOQ.FCT=="Decay") {

tryCatch({ #skip if model cannot be determined.

assign(paste0("LOQ.mod",i),nls(Cq.CV~SSasymp(log10(Standards),Asym,R0,lrc),

data=DAT2[DAT2$Target==Targets[i],]))

LOQ.list <- c(LOQ.list,paste0("LOQ.mod",i))

}, error=function(e) {

e

cat("ERROR: decay LOQ model cannot be defined for ",as.character(Targets[i]),sep="")

})

}

## Define LOQ model by linear modeling:

if(LOQ.FCT=="Linear") {

tryCatch({ #skip if model cannot be determined.

assign(paste0("LOQ.mod",i),lm(Cq.CV~log10(Standards),

data=DAT2[DAT2$Target==Targets[i],]))

LOQ.list <- c(LOQ.list,paste0("LOQ.mod",i))

}, error=function(e) {

e

cat("ERROR: linear LOQ model cannot be defined for ",as.character(Targets[i]),sep="")

})

}

## Define LOQ model by polynomial modeling:

if(substr(LOQ.FCT,1,1)=="P") {

Z <- as.numeric(substr(LOQ.FCT,2,nchar(LOQ.FCT)))

tryCatch({ #skip if model cannot be determined.

assign(paste0("LOQ.mod",i),lm(Cq.CV~poly(log10(Standards),Z),

data=DAT2[DAT2$Target==Targets[i],]))

LOQ.list <- c(LOQ.list,paste0("LOQ.mod",i))

}, error=function(e) {

e

cat("ERROR: ",Z,"-order polynomial LOQ model cannot be defined for ",

as.character(Targets[i]),sep="")

})

}

## Signal undetermined model with NA:

if(length(LOQ.list)<i+1) {

LOQ.list <- c(LOQ.list,NA)

}

## Define the logarithmic model for LOD using user-selected function:

if(is.list(LOD.FCT)==TRUE) {

tryCatch({ #skip if model cannot be determined.

assign(paste0("LOD.mod2",i),drm(Detect~SQ,data=DAT[DAT$Target==Targets[i],],fct=LOD.FCT))

LOD.list2 <- c(LOD.list2,paste0("LOD.mod2",i))

LOD.list3 <- c(LOD.list3,LOD.FCT$name)

}, error=function(e) {

e

cat("ERROR: LOD model cannot be defined for ",as.character(Targets[i]),sep="")

})

}

## Define the logarithmic model with function automatically selected:

if(is.character(LOD.FCT)) {

if(LOD.FCT=="Best") {

tryCatch({ #skip if model cannot be determined.

## Pull out data for specific assay:

TEMP.DAT <- DAT[DAT$Target==Targets[i],]

## Define a model to start with:

LOD.mod <- drm(Detect~SQ,data=TEMP.DAT,fct=W2.4())

## Test all available models and select the best one:

LOD.FCT2 <- row.names(mselect(LOD.mod,LOD.FCTS))[1]

LOD.FCT3 <- getMeanFunctions(fname=LOD.FCT2)

assign(paste0("LOD.mod2",i),drm(Detect~SQ,data=DAT[DAT$Target==Targets[i],],fct=LOD.FCT3[[1]]))

LOD.list2 <- c(LOD.list2,paste0("LOD.mod2",i))

LOD.list3 <- c(LOD.list3,LOD.FCT2)

}, error=function(e) {

e

cat("ERROR: LOD model cannot be defined for ",as.character(Targets[i]),sep="")

})

}

}

## Signal undetermined model with NA:

if(length(LOD.list2)<i+1) {

LOD.list2 <- c(LOD.list2,NA)

LOD.list3 <- c(LOD.list3,NA)

}

## Populate summary data:

DAT3$R.squared[i] <- summary(get(curve.list[i+1]))$r.squared

DAT3$Slope[i] <- coef(get(curve.list[i+1]))[2]

DAT3$Intercept[i] <- coef(get(curve.list[i+1]))[1]

DAT3$Low.95[i] <- min(DAT2$Standards[DAT2$Rate>=0.95&DAT2$Target==Targets[i]])

## Only get LOD values if the LOD model is defined:

if(!is.na(LOD.list2[i+1])) {

DAT3$LOD[i] <- ED(get(LOD.list2[i+1]),0.95,type="absolute")[1]

DAT3$rep2.LOD[i] <- ED(get(LOD.list2[i+1]),1-sqrt(0.05),type="absolute")[1]

DAT3$rep3.LOD[i] <- ED(get(LOD.list2[i+1]),1-0.05^(1/3),type="absolute")[1]

DAT3$rep4.LOD[i] <- ED(get(LOD.list2[i+1]),1-0.05^0.25,type="absolute")[1]

DAT3$rep5.LOD[i] <- ED(get(LOD.list2[i+1]),1-0.05^0.2,type="absolute")[1]

DAT3$rep8.LOD[i] <- ED(get(LOD.list2[i+1]),1-0.05^0.125,type="absolute")[1]

## Residual code using probit method:

#DAT3$LOD[i] <- (qnorm(0.95)-coef(get(LOD.list[i+1]))[1])/coef(get(LOD.list[i+1]))[2]

#DAT3$rep2.LOD[i] <- (qnorm(0.50)-coef(get(LOD.list[i+1]))[1])/coef(get(LOD.list[i+1]))[2]

#DAT3$rep3.LOD[i] <- (qnorm(1/3)-coef(get(LOD.list[i+1]))[1])/coef(get(LOD.list[i+1]))[2]

#DAT3$rep4.LOD[i] <- (qnorm(0.25)-coef(get(LOD.list[i+1]))[1])/coef(get(LOD.list[i+1]))[2]

#DAT3$rep5.LOD[i] <- (qnorm(0.2)-coef(get(LOD.list[i+1]))[1])/coef(get(LOD.list[i+1]))[2]

#DAT3$rep8.LOD[i] <- (qnorm(0.125)-coef(get(LOD.list[i+1]))[1])/coef(get(LOD.list[i+1]))[2]

}

## Generate prediction data for LoQ:

## Only get LOQ if LOQ model is determined:

if(!is.na(LOQ.list[i+1])) {

newData <- data.frame(Standards = seq(1, 10000))

newData$Cq.CV <- predict(get(LOQ.list[i+1]), newData)

## Determine what type of LOQ model is used and calculate LOQ accordingly:

## For exponential decay:

if(as.character(get(LOQ.list[i+1])$call)[1]=="nls") {

## Look up lowest modeled standard below the CV threshold:

DAT3$LOQ[i] <- min(newData$Standards[newData$Cq.CV<=LOQ.Threshold])

## Unless... If the background variation exceeds the CV threshold, adjust threshold:

## Determine the highest standard used:

A <- max(DAT2$Standards[DAT2$Target==Targets[i]])

if(min(newData$Cq.CV[newData$Standards<=A])>LOQ.Threshold) {

## Set the adjusted threshold to 1.5x the lowest simulated Cq.CV

## within the range of data tested:

B <- min(newData$Cq.CV[newData$Standards<=A])

DAT3$LOQ[i] <- min(newData$Standards[newData$Cq.CV<=B*1.5])

## Make a note of the adjusted threshold in the analysis log:

ToWrite <- paste0("Note: All standards tested for ",Targets[i],

" yielded higher Cq.CV values than the user-defined CV threshold of ",

LOQ.Threshold,". The CV threshold has been adjusted to ",

B*1.5," for the LOQ of this marker.")

write(paste0(ToWrite,"\n\n"),file="Analysis Log.txt",append=TRUE)

}

}

if(as.character(get(LOQ.list[i+1])$call)[1]=="lm") {

## For polynomial:

if(grepl("poly",as.character(get(LOQ.list[i+1])$call)[2])==TRUE) {

## Determine the highest standard used:

A <- max(DAT2$Standards[DAT2$Target==Targets[i]])

## Adjust if the tested range does not cross below the CV threshold:

if(min(DAT2$Cq.CV[DAT2$Target==Targets[i]],na.rm=TRUE)>LOQ.Threshold) {

B <- min(DAT2$Cq.CV[DAT2$Target==Targets[i]],na.rm=TRUE)*1.5

## Make a note of the adjusted threshold in the analysis log:

ToWrite <- paste0("Note: All standards tested for ",Targets[i],

" yielded higher Cq.CV values than the user-defined CV threshold of ",

LOQ.Threshold,". The CV threshold has been adjusted to ",

B," for the LOQ of this marker.")

write(paste0(ToWrite,"\n\n"),file="Analysis Log.txt",append=TRUE)

}

else {

B <- LOQ.Threshold

}

## Look up highest modeled standard below the CV threshold:

C <- max(newData$Standards[newData$Cq.CV<=B&newData$Standards<=A])

## Look up the highest modeled standard above the CV threshold...

## and also below the highest standard below the CV threshold.

## This captures the farthest right crossing point on a downward slope.

D <- max(newData$Standards[newData$Cq.CV>B&newData$Standards<C])

## LOQ is D + 1 to get back less than or equal to the CV threshold.

DAT3$LOQ[i] <- D+1

}

# For linear:

else {

## Look up lowest modeled standard below the CV threshold:

DAT3$LOQ[i] <- min(newData$Standards[newData$Cq.CV<=LOQ.Threshold])

}

}

## If modeled LOQ is calculated to be below the 95% LOD, set LOD as LOQ:

if(is.na(DAT3$LOD[i])==FALSE) {

if(DAT3$LOQ[i]<DAT3$LOD[i]) {

DAT3$LOQ[i] <- DAT3$LOD[i]

}

}

## If modeled LOQ is calculated to be below the lowest standard tested,

## set the lowest standard as the LOQ:

if(DAT3$LOQ[i]<min(DAT2$Standards[DAT2$Target==Targets[i]])) {

DAT3$LOQ[i] <- min(DAT2$Standards[DAT2$Target==Targets[i]])

}

}

}

write("Assay summary:",file="Analysis Log.txt",append=TRUE)

write("\nR.squared: The R-squared value of linear regression of all standards Cq-values vs log10 of the starting quantities.",file="Analysis Log.txt",append=TRUE)

write("Slope: The slope of the linear regression.",file="Analysis Log.txt",append=TRUE)

write("Intercept: The y-intercept of the linear regression.",file="Analysis Log.txt",append=TRUE)

write("\nLow.95: The lowest standard with at least 95% positive detection.",file="Analysis Log.txt",append=TRUE)

write("LOD: The 95% limit of detection as determined by probit modeling.",file="Analysis Log.txt",append=TRUE)

write(paste0("LOQ: The limit of quantification as determined by decay modeling, using the user-selected CV threshold of: ",LOQ.Threshold),file="Analysis Log.txt",append=TRUE)

write("\nrep2.LOD: The effective limit of detection if analyzing in 2 replicates.",file="Analysis Log.txt",append=TRUE)

write("rep3.LOD: The effective limit of detection if analyzing in 3 replicates.",file="Analysis Log.txt",append=TRUE)

write("rep4.LOD: The effective limit of detection if analyzing in 4 replicates.",file="Analysis Log.txt",append=TRUE)

write("rep5.LOD: The effective limit of detection if analyzing in 5 replicates.",file="Analysis Log.txt",append=TRUE)

write("rep8.LOD: The effective limit of detection if analyzing in 8 replicates.\n\n",file="Analysis Log.txt",append=TRUE)

write.csv(DAT3,file="Assay summary.csv",row.names=FALSE)

## Plot Cq value vs Standard Concentration standard curves:

DAT$Mod[DAT$Mod==0] <- "Excluded"

DAT$Mod[DAT$Mod==1] <- "Modeled"

for(i in 1:length(Targets)) {

ggOut <- ggplot(data=DAT[DAT$Target==Targets[i]&is.na(DAT$SQ)==FALSE,],

aes(x=SQ,y=Cq,color=factor(Mod),shape=factor(Mod),size=factor(Mod))) +

geom_jitter(width=0.1,alpha=0.75) +

scale_shape_manual("",values=c(3,20),guide=FALSE) +

scale_size_manual("",values=c(1,3)) +

scale_x_log10() +

scale_color_manual("",values=c("blue", "black")) +

xlab("Standard Concentrations (Copies / Reaction)") +

ylab("Cq-value") +

geom_abline(intercept=coef(get(curve.list[i+1]))[1],

slope=coef(get(curve.list[i+1]))[2]) +

geom_vline(xintercept=DAT3$LOD[i],colour="red") +

geom_vline(xintercept=DAT3$LOQ[i],linetype=2) +

annotate("text",y=max(DAT$Cq[DAT$Target==Targets[i]],na.rm=TRUE)*0.99,color="red",

x=DAT3$LOD[i]*0.8,angle=90,label="LOD") +

annotate("text",y=max(DAT$Cq[DAT$Target==Targets[i]],na.rm=TRUE)*0.94,

x=DAT3$LOQ[i]*0.8,angle=90,label="LOQ") +

theme_bw() + theme(legend.justification=c(1,1),legend.position=c(1,0.99)) +

ggtitle(paste0("Standard curve for: ",Targets[i])) +

theme(plot.title=element_text(hjust=0.5,size=20),

axis.title=element_text(size=16)) +

theme(legend.title=element_blank(),

legend.text=element_text(size=11)) +

annotate("text",y=min(DAT$Cq[DAT$Target==Targets[i]&is.na(DAT$SQ)==FALSE],na.rm=TRUE)*1.05,

x=min(DAT$SQ[DAT$Target==Targets[i]&is.na(DAT$SQ)==FALSE],na.rm=TRUE)*1.01,hjust=0,

label=(paste0("R-squared: ",DAT3$R.squared[i],"\ny = ",DAT3$Slope[i],"x + ",DAT3$Intercept[i])))

print(ggOut)

readline(prompt="Press [Enter] for next plot.")

print("Calculating... Please wait.")

}

## Plot the LOD models for each assay:

for(i in 1:length(Targets)) {

if(!is.na(LOD.list2[i+1])) {

DAT4 <- rbind(ED(get(LOD.list2[i+1]),0.95,interval="delta",type="absolute"),

ED(get(LOD.list2[i+1]),1-sqrt(0.05),interval="delta",type="absolute"),

ED(get(LOD.list2[i+1]),1-0.05^(1/3),interval="delta",type="absolute"),

ED(get(LOD.list2[i+1]),1-0.05^0.25,interval="delta",type="absolute"),

ED(get(LOD.list2[i+1]),1-0.05^0.2,interval="delta",type="absolute"),

ED(get(LOD.list2[i+1]),1-0.05^0.125,interval="delta",type="absolute"))

if(substr(LOD.list3[i+1],1,3)=="LL2") {

DAT4 <- exp(DAT4)

}

DAT4 <- data.frame(DAT4,LoD=c("1rep.LOD","2rep.LOD","3rep.LOD","4rep.LOD",

"5rep.LOD","8rep.LOD"),

Assay=rep(Targets[i],nrow(DAT4)))

DAT4$Assay <- as.character(DAT4$Assay)

if(i==1) {

LOD.CI <- DAT4

}

if(i>1) {

LOD.CI <- rbind(LOD.CI,DAT4)

}

if(sum(!is.na(DAT4[,3])&DAT4[,3]<=0)>0) { #Unable to plot negative lower limits, converting any lower limit values to 0.0001

DAT4[!is.na(DAT4[,3])&DAT4[,3]<=0,3] <- 0.0001

}

plot(get(LOD.list2[i+1]),main=paste0("LoD Plot for: ",Targets[i]),

ylab="Detection Probability",xlab="Standard concentrations (Copies / Reaction)",

xlim=c(min(DAT4[,1:4],na.rm=TRUE),max(DAT$SQ,na.rm=TRUE)))

LODS <- sum(!is.na(DAT4[,1]))

COLS <- c(rgb(0.8,0.47,0.65),rgb(0,0.45,0.7),rgb(0.94,0.89,0.26),

rgb(0.84,0.37,0),rgb(0,0.62,0.45),rgb(0.90,0.62,0))

PNTS <- c(15,16,17,18,25,3)

YS <- c(0.95,1-sqrt(0.05),1-0.05^(1/3),1-0.05^0.25,1-0.05^0.2,1-0.05^0.125)

LODS2 <- c("Limit of Detection","2 Replicates LoD","3 Replicates LoD",

"4 Replicates LoD","5 Replicates LoD","8 Replicates LoD")

if(LODS<6) {

LODS2[(LODS+1):6] <- gsub("licates LoD","s: Insufficient Data",LODS2[(LODS+1):6])

}

points(x=DAT4[1:LODS,1],y=YS[1:LODS],pch=PNTS,col=COLS,cex=1.2)

for(j in 1:LODS) {

lines(x=DAT4[j,3:4],y=rep(YS[j],2),col=COLS[j],lwd=2)

lines(x=rep(DAT4[j,3],2),y=c(YS[j]-0.02,YS[j]+0.02),lwd=2,col=COLS[j])

lines(x=rep(DAT4[j,4],2),y=c(YS[j]-0.02,YS[j]+0.02),lwd=2,col=COLS[j])

}

legend("bottomright",legend=LODS2,pch=PNTS,col=COLS,text.col=COLS)

Pval <- modelFit(get(LOD.list2[i+1]))[[5]][2]

mtext(paste0("FCT used: ",LOD.list3[i+1]," Lack of fit test: p = ",Pval),side=3)

}

if(is.na(LOD.list2[i+1])) {

plot(DAT2$Rate[DAT2$Target==Targets[i]]~log10(DAT2$Standards[DAT2$Target==Targets[i]]),

ylim=c(0,1),ylab="Detection Probability",

xlab=expression("Log of standard concentrations (Log"[10]*"Copies / Reaction)"),

main=paste0("LoD for: ",Targets[i]," unsolvable"))

}

readline(prompt="Press [Enter] for next plot.")

print("Calculating... Please wait.")

}

LOD.CI <- LOD.CI[,c(6,5,1,3,4,2)]

write.csv(LOD.CI,file="LOD_confint.csv",row.names=FALSE)

## Plot the LoQ models of each assay:

for(i in 1:length(Targets)) {

if(is.na(LOQ.list[i+1])==FALSE) {

## Re-generate prediction data for the model:

newData <- data.frame(Standards = seq(1, 10000))

newData$Cq.CV <- predict(get(LOQ.list[i+1]), newData)

## Define LOQ polygon coordinates:

PDAT <- data.frame(x=c(min(DAT2$Standards[DAT2$Target==Targets[i]],na.rm=TRUE),

min(DAT2$Standards[DAT2$Target==Targets[i]],na.rm=TRUE),

DAT3$LOQ[DAT3$Assay==Targets[i]],

DAT3$LOQ[DAT3$Assay==Targets[i]]),

y=c(min(c(DAT2$Cq.CV[DAT2$Target==Targets[i]],newData$Cq.CV[newData$Standards<=max(DAT2$Standards[DAT2$Target==Targets[i]])&newData$Standards>=min(DAT2$Standards[DAT2$Target==Targets[i]])]),na.rm=TRUE)*0.9,

newData$Cq.CV[newData$Standards==DAT3$LOQ[DAT3$Assay==Targets[i]]],

newData$Cq.CV[newData$Standards==DAT3$LOQ[DAT3$Assay==Targets[i]]],

min(c(DAT2$Cq.CV[DAT2$Target==Targets[i]],newData$Cq.CV[newData$Standards<=max(DAT2$Standards[DAT2$Target==Targets[i]])&newData$Standards>=min(DAT2$Standards[DAT2$Target==Targets[i]])]),na.rm=TRUE)*0.9))

if(DAT3$LOQ[DAT3$Assay==Targets[i]]!=floor(DAT3$LOQ[DAT3$Assay==Targets[i]])) {

PDAT$y[2:3] <- LOQ.Threshold

}

}

Decay.Plot <- ggplot(DAT2[DAT2$Target==Targets[i],], aes(x= Standards, y = Cq.CV)) +

geom_point(size=2) +

scale_x_continuous(trans = 'log10') +

ylab("Coefficient of variation for Cq-Values") +

xlab("Standard concentrations (Copies / Reaction)") +

geom_vline(xintercept=DAT3$LOD[DAT3$Assay==Targets[i]],color="red") +

annotate("text",y=max(DAT2$Cq.CV[DAT2$Target==Targets[i]],na.rm=TRUE)*0.99,

x=DAT3$LOD[i]*0.8,angle=90,label="LOD",color="red") +

theme(legend.position="none") +

theme(plot.title=element_text(hjust=0.5))

if(is.na(LOQ.list[i+1])==FALSE) {

if(DAT3$LOQ[DAT3$Assay==Targets[i]]<=min(DAT2$Standards[DAT2$Target==Targets[i]])) {

PDAT$x[3:4] <- NA

Decay.Plot <- Decay.Plot +

annotate("text",y=max(DAT2$Cq.CV[DAT2$Target==Targets[i]],na.rm=TRUE)*0.99,

x=median(DAT2$Standards[DAT2$Target==Targets[i]]),

label="LOQ may be outside tested range.",hjust=0)

}

Decay.Plot <- Decay.Plot + geom_polygon(data=PDAT,aes(x=x,y=y,alpha=0.5))

if(as.character(get(LOQ.list[i+1])$call)[1]=="nls") {

Decay.Plot <- Decay.Plot +

stat_smooth(method = "nls", formula = y ~ SSasymp(x, Asym, R0, lrc), se = FALSE) +

ggtitle(paste0("Exponential Decay LOQ model for: ",Targets[i]))

}

if(as.character(get(LOQ.list[i+1])$call)[1]=="lm") {

if(grepl("poly",as.character(get(LOQ.list[i+1])$call)[2])==TRUE) {

B <- length(get(LOQ.list[i+1])$coefficients)-1

Decay.Plot <- Decay.Plot +

stat_smooth(method = "lm", formula = y ~ poly(x,B),se=FALSE) +

ggtitle(paste0(B,"-order polynomial LOQ model for: ",Targets[i]))

}

else {

Decay.Plot <- Decay.Plot +

stat_smooth(method = "lm", formula = y ~ x,se=FALSE) +

ggtitle(paste0("Linear LOQ model for: ",Targets[i]))

}

}

}

if(is.na(LOQ.list[i+1])==TRUE) {

Decay.Plot <- Decay.Plot +

ggtitle(paste0("LOQ model for: ",Targets[i]," not solvable."))

}

print(Decay.Plot)

readline(prompt="Press [Enter] for next plot.")

print("Calculating... Please wait.")

}

#**Figure S2** Limit of Detection estimation for qPCR. Figure and calculations were performed following the method outlined in Hunter ME, Dorazio RM, Butterfield JSS, Meigs-Friend G, Nico LG, Ferrante JA (2017) Detection limits of quantitative and digital PCR assays and their influence in presence–absence surveys of environmental DNA. Mol Ecol Resour 17:221–229. https://doi.org/10.1111/1755-0998.12619

### Definition of function used to maximize log-likelihood of linear model with non-constant variance

negLL = function(param, y, X, Z) {

beta = param[1:3]

sigma = exp(param[-(1:3)])

muVec = as.vector(X %*% beta)

sigmaVec = as.vector(Z %*% sigma)

logL = dnorm(y, mean=muVec, sd=sigmaVec, log=TRUE)

(-1)*sum(logL)

}

### Definition of function used to estimate standard curve and limit of detection

LODmodel.lm = function(xminOfStds, y, x) {

## partition observations into standards and non-standards

std = x>=xminOfStds

y.low = y[!std]

x.low = x[!std]

y.std = y[std]

x.std = x[std]

## fit model to standards

fit = glm(y.std~log(x.std), family=gaussian)

beta.mle = fit$coefficients

## fit model to non-standards

fit.low = glm(y.low~1, family=gaussian)

alpha.mle = fit.low$coefficients

## fit model to all data

x1 = matrix(as.integer(std), nrow=length(y))

x2 = matrix(0, nrow=length(y))

x2[std] = log(x[std])

X = cbind(x1, x2, x3=1-x1)

Z = model.matrix(~factor(x)-1)

sigma.mle = tapply(y, factor(x), sd)

paramGuess = c(beta.mle, alpha.mle, log(sigma.mle))

fit.glm = optim(par=paramGuess, fn=negLL, method='BFGS', hessian=TRUE, y=y, X=X, Z=Z)

fit.glm.aic = 2*fit.glm$value + 2*length(fit$par)

mle = fit.glm$par

vcov = matrix(nrow=length(paramGuess), ncol=length(paramGuess))

if (all( eigen(fit.glm$hessian)$values>0 ) ) {

vcov = chol2inv(chol(fit.glm$hessian))

}

vcv = vcov[1:3, 1:3]

## compute estimate of limit of detection

x.LOD = (mle[3] - mle[1]) / mle[2]

x.LOD.var = (vcv[3,3] + vcv[1,1] + x.LOD*x.LOD*vcv[2,2] - 2*vcv[3,1] - 2*x.LOD*vcv[3,2] + 2*x.LOD*vcv[1,2]) / (mle[2]*mle[2])

x.LOD.se = sqrt(x.LOD.var)

list(beta.mle=mle[1:2], beta.se=sqrt(diag(vcv[1:2,1:2])), alpha.mle=mle[3], alpha.se=sqrt(vcv[3,3]), logLOD=unname(x.LOD), logLOD.se=unname(x.LOD.se), aic=fit.glm.aic)

}

### Definition of function used to print estimates of model parameters and limit of detection (LOD)

LODmodel.lm.summary = function(fit) {

zcrit = qnorm(1-0.05/2)

beta.lowerCL = fit$beta.mle - zcrit*fit$beta.se

beta.upperCL = fit$beta.mle + zcrit*fit$beta.se

alpha.lowerCL = fit$alpha.mle - zcrit*fit$alpha.se

alpha.upperCL = fit$alpha.mle + zcrit*fit$alpha.se

logLOD.lowerCL = fit$logLOD - zcrit*fit$logLOD.se

logLOD.upperCL = fit$logLOD + zcrit*fit$logLOD.se

out = cbind(fit$beta.mle, fit$beta.se, beta.lowerCL, beta.upperCL)

out = rbind(out, c(fit$alpha.mle, fit$alpha.se, alpha.lowerCL, alpha.upperCL))

out = rbind(out, c(fit$logLOD, fit$logLOD.se, logLOD.lowerCL, logLOD.upperCL))

out = rbind(out, c(exp(fit$logLOD), NA, exp(logLOD.lowerCL), exp(logLOD.upperCL)))

dimnames(out)[1] = list(c('beta0','beta1', 'alpha', 'logLOD', 'LOD'))

dimnames(out)[2] = list(c('MLE', 'SE', '2.5%', '97.5%'))

print(out)

}

### Definitions of variables:

## y = vector of complement of cycle threshold (= maximum possible Ct - Ct)

## x = vector of standard concentration (copies per uL) in PCR reaction chamber

### Input qPCR data for serial dilution experiment

d = read.csv(file='LB_qPCR_synth_gene_dil.csv', na.strings='Undetermined')

sample = as.character(d[,'Sample'])

ind = sample != 'a'

x.sample = d[ind,'Expected_stand_conc']

#x.sample <- as.numeric(x.sample)

x = x.sample * 1 / 20 # 1 uL of sample in total volume of 20 uL

CycleThreshold = d[ind,'Ct']

maxCycleThreshold = 40 #number of cycles used in qPCR

CycleThreshold[is.na(CycleThreshold)] = maxCycleThreshold

y = maxCycleThreshold - CycleThreshold

### Evaluate AIC (Akaike's information criterion) for each standard concentration

x.unique = sort(unique(x))

nstds = length(x.unique)

x.unique = x.unique[2:(nstds-1)]

aic = rep(NA, length(x.unique))

for (i in 1:length(x.unique)) {

fit.LODmodel = LODmodel.lm(x.unique[i], y=y, x=x)

aic[i] = fit.LODmodel$aic

}

print(cbind(Conc=x.unique, AIC=aic))

### Estimate parameters of standard curve and limit of detection

ind = aic==min(aic)

xValue = x.unique[ind]

fit = LODmodel.lm(xValue, y=y, x=x)

LODmodel.lm.summary(fit)

### Plot estimated concentrations vs. standard concentrations

ymean = tapply(y, x, mean, na.rm=TRUE)

x.unique = sort(unique(x))

par(oma=c(3,5,0.5,0.5), mar=c(3,3,0.5,0.5))

xlim = c(-6, 9)

ylim = c(0, 28)

plot(log(x), y, cex=1.5, lwd=2, xlim=xlim, xlab='', ylab='', las=1, cex.axis=1.4)

points(log(x.unique), ymean, cex=2, lwd=2, pch='x', col='red')

zcrit = qnorm(1-0.05/2)

logLOD.lowerCL = fit$logLOD - zcrit*fit$logLOD.se

logLOD.upperCL = fit$logLOD + zcrit*fit$logLOD.se

lines(rep(fit$logLOD,2), c(ylim[1]-1, fit$beta.mle[1]+fit$beta.mle[2]*fit$logLOD), lty=2, lwd=2, col='blue')

lines(c(xlim[1]-1, fit$logLOD), rep(fit$beta.mle[1]+fit$beta.mle[2]*fit$logLOD,2), lty=2, lwd=2, col='blue')

lines(rep(logLOD.upperCL,2), c(ylim[1]-1, fit$beta.mle[1]+fit$beta.mle[2]*logLOD.upperCL), lty=3, lwd=2, col='blue')

lines(rep(logLOD.lowerCL,2), c(ylim[1]-1, fit$beta.mle[1]+fit$beta.mle[2]*logLOD.lowerCL), lty=3, lwd=2, col='blue')

lines(c(xlim[1]-1, logLOD.upperCL), rep(fit$beta.mle[1]+fit$beta.mle[2]*logLOD.upperCL,2), lty=3, lwd=2, col='blue')

lines(c(xlim[1]-1, logLOD.lowerCL), rep(fit$beta.mle[1]+fit$beta.mle[2]*logLOD.lowerCL,2), lty=3, lwd=2, col='blue')

lines(c(fit$logLOD, xlim[2]+1), fit$beta.mle[1] + fit$beta.mle[2]*c(fit$logLOD, xlim[2]+1), lty=1, lwd=2, col='black')

mtext(expression(paste('Log of concentration (copies / ', mu, 'L)')), side=1, cex=1.8, outer=TRUE)

mtext('Complement of cycle threshold', side=2, cex=1.8, line=4, outer=FALSE)

#**Figure S3** Limit of detection estimation for dPCR. Figure and calculations were performed following the method outlined in Hunter ME, Dorazio RM, Butterfield JSS, Meigs-Friend G, Nico LG, Ferrante JA (2017) Detection limits of quantitative and digital PCR assays and their influence in presence–absence surveys of environmental DNA. Mol Ecol Resour 17:221–229. https://doi.org/10.1111/1755-0998.12619

### Definition of function used to estimate standard curve and limit of detection

LODmodel.glm = function(xminOfStds, y, m, x, v=0.91/1000) {

## partition observations into standards and non-standards

std = x>=xminOfStds

y.low = y[!std]

m.low = m[!std]

x.low = x[!std]

y.std = y[std]

m.std = m[std]

x.std = x[std]

## fit model to standards

v.offset = rep(log(v), length(y.std))

ymat = cbind(y.std, m.std-y.std)

fit = glm(ymat~log(x.std), family=binomial(link='cloglog'), offset=v.offset)

beta.mle = fit$coefficients

## fit model to non-standards

v.offset = rep(log(v), length(y.low))

ymat = cbind(y.low, m.low-y.low)

fit.low = glm(ymat~1, family=binomial(link='cloglog'), offset=v.offset)

alpha.mle = fit.low$coefficients

## fit model to all data

x1 = matrix(as.integer(std), nrow=length(y))

x2 = matrix(0, nrow=length(y))

x2[std] = log(x[std])

X = cbind(x1, x2, x3=1-x1)

v.offset = rep(log(v), length(y))

ymat = cbind(y, m-y)

fit.glm = glm(ymat~X-1, family=binomial(link='cloglog'), offset=v.offset)

fit.glm.aic = fit.glm$aic

mle = fit.glm$coeff

vcv = vcov(fit.glm)

## compute estimate of limit of detection

x.LOD = (mle[3] - mle[1]) / mle[2]

x.LOD.var = (vcv[3,3] + vcv[1,1] + x.LOD*x.LOD*vcv[2,2] - 2*vcv[3,1] - 2*x.LOD*vcv[3,2] + 2*x.LOD*vcv[1,2]) / (mle[2]*mle[2])

x.LOD.se = sqrt(x.LOD.var)

list(beta.mle=mle[1:2], beta.se=sqrt(diag(vcv[1:2,1:2])), alpha.mle=mle[3], alpha.se=sqrt(vcv[3,3]), logLOD=unname(x.LOD), logLOD.se=unname(x.LOD.se), aic=fit.glm.aic)

}

### Definition of function used to print estimates of model parameters and limit of detection (LOD)

LODmodel.glm.summary = function(fit) {

zcrit = qnorm(1-0.05/2)

beta.lowerCL = fit$beta.mle - zcrit*fit$beta.se

beta.upperCL = fit$beta.mle + zcrit*fit$beta.se

alpha.lowerCL = fit$alpha.mle - zcrit*fit$alpha.se

alpha.upperCL = fit$alpha.mle + zcrit*fit$alpha.se

logLOD.lowerCL = fit$logLOD - zcrit*fit$logLOD.se

logLOD.upperCL = fit$logLOD + zcrit*fit$logLOD.se

out = cbind(fit$beta.mle, fit$beta.se, beta.lowerCL, beta.upperCL)

out = rbind(out, c(fit$alpha.mle, fit$alpha.se, alpha.lowerCL, alpha.upperCL))

out = rbind(out, c(fit$logLOD, fit$logLOD.se, logLOD.lowerCL, logLOD.upperCL))

out = rbind(out, c(exp(fit$logLOD), NA, exp(logLOD.lowerCL), exp(logLOD.upperCL)))

dimnames(out)[1] = list(c('beta0','beta1', 'alpha', 'logLOD', 'LOD'))

dimnames(out)[2] = list(c('MLE', 'SE', '2.5%', '97.5%'))

print(out)

}

### Definitions of variables:

## y = vector of number of positive partitions

## m = vector of total number of partitions

## x = vector of standard concentration (copies per uL) in PCR reaction chamber

## v = constant volume (uL) of each partition

### Input dPCR data for serial dilution experiment####

d = read.table(file='LB_dPCR_synth_gene_dil95.csv', header=TRUE, sep = ",")

y = d[,'npositive']

m = d[,'ntotal']

x.sample = d[,'SampleConc']

x = x.sample * 1 / 10 # 1 uL of sample in total volume of 10 uL

v = 0.91 / 1000 # constant volume (microliters) per droplet

### Evaluate AIC (Akaike's information criterion) for each standard concentration

x.unique = sort(unique(x))

nstds = length(x.unique)

x.unique = x.unique[2:(nstds-1)]

aic = rep(NA, length(x.unique))

for (i in 1:length(x.unique)) {

fit.LODmodel = LODmodel.glm(x.unique[i], y=y, m=m, x=x)

aic[i] = fit.LODmodel$aic

}

print(cbind(Conc=x.unique, AIC=aic))

### Estimate parameters of standard curve and limit of detection

ind = aic==min(aic)

xValue = x.unique[ind]

fit = LODmodel.glm(xValue, y=y, m=m, x=x)

LODmodel.glm.summary(fit)

### Plot estimated concentrations vs. standard concentrations

lambda.repl = (-1/v) * log(1 - y/m)

ysum = tapply(y, x, sum)

msum = tapply(m, x, sum)

lambda.sum = (-1/v) * log(1 - ysum/msum)

x.unique = sort(unique(x))

par(oma=c(3,5,0.5,0.5), mar=c(3,3,0.5,0.5))

xylim = c(-5,8)

xylim = c(-6,9)

plot(log(x), log(lambda.repl), cex=1.5, lwd=2, xlim=xylim, ylim=xylim, xlab='', ylab='', las=1, cex.axis=1.4)

points(log(x.unique), log(lambda.sum), cex=2, lwd=2, pch='x', col='red')

zcrit = qnorm(1-0.05/2)

logLOD.lowerCL = fit$logLOD - zcrit*fit$logLOD.se

logLOD.upperCL = fit$logLOD + zcrit*fit$logLOD.se

lines(rep(fit$logLOD,2), c(xylim[1]-1, fit$logLOD), lty=2, lwd=2, col='blue')

lines(c(xylim[1]-1, fit$logLOD), rep(fit$logLOD,2), lty=2, lwd=2, col='blue')

lines(rep(logLOD.upperCL,2), c(xylim[1]-1, logLOD.upperCL), lty=3, lwd=2, col='blue')

lines(rep(logLOD.lowerCL,2), c(xylim[1]-1, logLOD.lowerCL), lty=3, lwd=2, col='blue')

lines(c(xylim[1]-1, logLOD.upperCL), rep(logLOD.upperCL,2), lty=3, lwd=2, col='blue')

lines(c(xylim[1]-1, logLOD.lowerCL), rep(logLOD.lowerCL,2), lty=3, lwd=2, col='blue')

lines(c(xylim[1]-1, fit$logLOD), rep(fit$alpha.mle,2), lty=1, lwd=2, col='black')

lines(c(fit$logLOD, xylim[2]+1), fit$beta.mle[1] + fit$beta.mle[2]*c(fit$logLOD, xylim[2]+1), lty=1, lwd=2, col='black')

mtext(expression(paste('Log of concentration (copies / ', mu, 'L)')), side=1, cex=1.8, outer=TRUE)

mtext(expression(paste('Log of measured concentration (copies / ', mu, 'L)')), side=2, cex=1.8, line=4, outer=FALSE)

# **Figure S4.** Limit of Detection estimation for ddPCR. Figure and calculations were performed following the method outlined in Hunter ME, Dorazio RM, Butterfield JSS, Meigs-Friend G, Nico LG, Ferrante JA (2017) Detection limits of quantitative and digital PCR assays and their influence in presence–absence surveys of environmental DNA. Mol Ecol Resour 17:221–229. <https://doi.org/10.1111/1755-0998.12619>

### Definition of function used to estimate standard curve and limit of detection

LODmodel.glm = function(xminOfStds, y, m, x, v=0.91/1000) {

## partition observations into standards and non-standards

std = x>=xminOfStds

y.low = y[!std]

m.low = m[!std]

x.low = x[!std]

y.std = y[std]

m.std = m[std]

x.std = x[std]

## fit model to standards

v.offset = rep(log(v), length(y.std))

ymat = cbind(y.std, m.std-y.std)

fit = glm(ymat~log(x.std), family=binomial(link='cloglog'), offset=v.offset)

beta.mle = fit$coefficients

## fit model to non-standards

v.offset = rep(log(v), length(y.low))

ymat = cbind(y.low, m.low-y.low)

fit.low = glm(ymat~1, family=binomial(link='cloglog'), offset=v.offset)

alpha.mle = fit.low$coefficients

## fit model to all data

x1 = matrix(as.integer(std), nrow=length(y))

x2 = matrix(0, nrow=length(y))

x2[std] = log(x[std])

X = cbind(x1, x2, x3=1-x1)

v.offset = rep(log(v), length(y))

ymat = cbind(y, m-y)

fit.glm = glm(ymat~X-1, family=binomial(link='cloglog'), offset=v.offset)

fit.glm.aic = fit.glm$aic

mle = fit.glm$coeff

vcv = vcov(fit.glm)

## compute estimate of limit of detection

x.LOD = (mle[3] - mle[1]) / mle[2]

x.LOD.var = (vcv[3,3] + vcv[1,1] + x.LOD*x.LOD*vcv[2,2] - 2*vcv[3,1] - 2*x.LOD*vcv[3,2] + 2*x.LOD*vcv[1,2]) / (mle[2]*mle[2])

x.LOD.se = sqrt(x.LOD.var)

list(beta.mle=mle[1:2], beta.se=sqrt(diag(vcv[1:2,1:2])), alpha.mle=mle[3], alpha.se=sqrt(vcv[3,3]), logLOD=unname(x.LOD), logLOD.se=unname(x.LOD.se), aic=fit.glm.aic)

}

### Definition of function used to print estimates of model parameters and limit of detection (LOD)

LODmodel.glm.summary = function(fit) {

zcrit = qnorm(1-0.05/2)

beta.lowerCL = fit$beta.mle - zcrit*fit$beta.se

beta.upperCL = fit$beta.mle + zcrit*fit$beta.se

alpha.lowerCL = fit$alpha.mle - zcrit*fit$alpha.se

alpha.upperCL = fit$alpha.mle + zcrit*fit$alpha.se

logLOD.lowerCL = fit$logLOD - zcrit*fit$logLOD.se

logLOD.upperCL = fit$logLOD + zcrit*fit$logLOD.se

out = cbind(fit$beta.mle, fit$beta.se, beta.lowerCL, beta.upperCL)

out = rbind(out, c(fit$alpha.mle, fit$alpha.se, alpha.lowerCL, alpha.upperCL))

out = rbind(out, c(fit$logLOD, fit$logLOD.se, logLOD.lowerCL, logLOD.upperCL))

out = rbind(out, c(exp(fit$logLOD), NA, exp(logLOD.lowerCL), exp(logLOD.upperCL)))

dimnames(out)[1] = list(c('beta0','beta1', 'alpha', 'logLOD', 'LOD'))

dimnames(out)[2] = list(c('MLE', 'SE', '2.5%', '97.5%'))

print(out)

}

### Definitions of variables:

## y = vector of number of positive partitions

## m = vector of total number of partitions

## x = vector of standard concentration (copies per uL) in PCR reaction chamber

## v = constant volume (uL) of each partition

### Input dPCR data for serial dilution experiment ####

d = read.table(file='LB_ddPCR_synth_gene_dil95.csv', header=TRUE, sep = ",")

y = d[,'npositive']

m = d[,'ntotal']

x.sample = d[,'SampleConc']

x = x.sample * 1 / 20 # 1 uL of sample in total volume of 20 uL

v = 0.91 / 1000 # constant volume (microliters) per droplet

### Evaluate AIC (Akaike's information criterion) for each standard concentration

x.unique = sort(unique(x))

nstds = length(x.unique)

x.unique = x.unique[2:(nstds-1)]

aic = rep(NA, length(x.unique))

for (i in 1:length(x.unique)) {

fit.LODmodel = LODmodel.glm(x.unique[i], y=y, m=m, x=x)

aic[i] = fit.LODmodel$aic

}

print(cbind(Conc=x.unique, AIC=aic))

### Estimate parameters of standard curve and limit of detection

ind = aic==min(aic)

xValue = x.unique[ind]

fit = LODmodel.glm(xValue, y=y, m=m, x=x)

LODmodel.glm.summary(fit)

### Plot estimated concentrations vs. standard concentrations

lambda.repl = (-1/v) * log(1 - y/m)

ysum = tapply(y, x, sum)

msum = tapply(m, x, sum)

lambda.sum = (-1/v) * log(1 - ysum/msum)

x.unique = sort(unique(x))

par(oma=c(3,5,0.5,0.5), mar=c(3,3,0.5,0.5))

xylim = c(-5,8)

xylim = c(-6,9)

plot(log(x), log(lambda.repl), cex=1.5, lwd=2, xlim=xylim, ylim=xylim, xlab='', ylab='', las=1, cex.axis=1.4)

points(log(x.unique), log(lambda.sum), cex=2, lwd=2, pch='x', col='red')

zcrit = qnorm(1-0.05/2)

logLOD.lowerCL = fit$logLOD - zcrit*fit$logLOD.se

logLOD.upperCL = fit$logLOD + zcrit*fit$logLOD.se

lines(rep(fit$logLOD,2), c(xylim[1]-1, fit$logLOD), lty=2, lwd=2, col='blue')

lines(c(xylim[1]-1, fit$logLOD), rep(fit$logLOD,2), lty=2, lwd=2, col='blue')

lines(rep(logLOD.upperCL,2), c(xylim[1]-1, logLOD.upperCL), lty=3, lwd=2, col='blue')

lines(rep(logLOD.lowerCL,2), c(xylim[1]-1, logLOD.lowerCL), lty=3, lwd=2, col='blue')

lines(c(xylim[1]-1, logLOD.upperCL), rep(logLOD.upperCL,2), lty=3, lwd=2, col='blue')

lines(c(xylim[1]-1, logLOD.lowerCL), rep(logLOD.lowerCL,2), lty=3, lwd=2, col='blue')

lines(c(xylim[1]-1, fit$logLOD), rep(fit$alpha.mle,2), lty=1, lwd=2, col='black')

lines(c(fit$logLOD, xylim[2]+1), fit$beta.mle[1] + fit$beta.mle[2]*c(fit$logLOD, xylim[2]+1), lty=1, lwd=2, col='black')

mtext(expression(paste('Log of concentration (copies / ', mu, 'L)')), side=1, cex=1.8, outer=TRUE)

mtext(expression(paste('Log of measured concentration (copies / ', mu, 'L)')), side=2, cex=1.8, line=4, outer=FALSE)

# **Figure S5.** Limit of Quantification (LOQ) of dPCR.

############## LOQ ##########

# Load the dplyr package

library(dplyr)

d = read.table(file='LB_dPCR_synth_gene_dil95.csv', header=TRUE, sep = ",")

# Calculate CV, mean, and SD for each EstConc

d_summary <- d %>%

group_by(SampleConc) %>%

summarize(

CV = sd(EstConc) / mean(EstConc) * 100,

mean_EstConc = mean(EstConc),

sd_EstConc = sd(EstConc)

)

# Print the summary

print(d_summary)

# Load the ggplot2 package

library(ggplot2)

# Filter out the two lowest and the highest sample concentrations

dPCR_summary_filtered <- d_summary %>%

filter(SampleConc > 0.488 & SampleConc < 48795.1)

# x = x.sample * 1 / 10 # 1 uL of sample in total volume of 10 uL

# Calculate x_mean_estconc to account for volume

dPCR_summary_filtered <- dPCR_summary_filtered %>%

mutate(x_mean_estconc = mean_EstConc * 1 / 10)

# View the modified data frame

print(dPCR_summary_filtered)

y=dPCR_summary_filtered$CV

x=dPCR_summary_filtered$x_mean_estconc

summary(lm(y~ log(x)))

exp((35-50.055)/-19.961)

#2.125954

LOQ_dPCRtransf<- ggplot(dPCR_summary_filtered, aes(x = (x_mean_estconc), y = CV)) +

geom_point() +

geom_smooth(method = "lm", formula = y ~ log(x), se = FALSE, color = "#496C7A") + # Add logarithmic curve

geom_hline(yintercept = 35, linetype = "dashed", color = "#A7473A") + # Add horizontal line for CV = 35

labs(x = "Concentration (copies/ µL)", y = "Coefficient of Variation (%)", title = "dPCR") +

scale_x_continuous(breaks = seq(0, 10))+

#scale_y_continuous(breaks = seq(-0, 160, 20))+

geom_vline(xintercept = 2.125954, linetype = "dashed", color = "#009E73")+

geom_point(x = 2.125954, y = 35, color = "maroon", size = 5)+ # Add the point at x = 7

geom_text(aes(label = 'LOQ=2.12'), x = 3, y = 36, vjust = -0.5, hjust = 1, color = "black", size = 4)+

theme_classic()+

theme(axis.text.x = element_text(angle = 0, vjust = 0.5, hjust = 1, size = 12),

axis.text.y = element_text(size = 12),

axis.title = element_text(size = 14),

legend.text = element_text(size = 12))

LOQ_dPCRtransf

#**Figure S6.** Limit of Quantification (LOQ) of ddPCR.

##LOQ for ddPCR results

# Load the dplyr package

library(dplyr)

#Load the ggplot2 package

library(ggplot2)

#### ddPCR #####

d = read.table(file='LB_ddPCR_synth_gene_dil95.csv', header=TRUE, sep = ",")

# Calculate CV, mean, and SD for each EstConc

d_summary <- d %>%

group_by(SampleConc) %>%

summarize(

CV = sd(EstConc) / mean(EstConc) * 100,

mean_EstConc = mean(EstConc),

sd_EstConc = sd(EstConc)

)

# Print the summary

print(d_summary)

# Filter out the two lowest and the highest sample concentrations

ddPCR_summary_filtered <- d_summary %>%

filter(SampleConc > 0.488 & SampleConc < 48795.1)

print(ddPCR_summary_filtered)

# Calculate x_mean_estconc to account for volume

ddPCR_summary_filtered <- ddPCR_summary_filtered %>%

mutate(x_mean_estconc = mean_EstConc * 1 / 20)

y=ddPCR_summary_filtered$CV

x=ddPCR_summary_filtered$x_mean_estconc

summary(lm(y~ log(x)))

exp((35-42.02)/-32.80)

# 1.23

LOQ<- ggplot(ddPCR_summary_filtered, aes(x = (x_mean_estconc), y = CV)) +

geom_point() +

geom_smooth(method = "lm", formula = y ~ log(x), se = FALSE, color = "#496C7A") + # Add logarithmic curve

geom_hline(yintercept = 35, linetype = "dashed", color = "#A7473A") + # Add horizontal line for CV = 35

labs(x = "Concentration (copies/µL)", y = "Coefficient of Variation (%)", title = "ddPCR") +

# scale_x_continuous(breaks = seq(0, 40, 10))+

scale_y_continuous(breaks = seq(-20, 160, 20))+

geom_vline(xintercept = 1.23, linetype = "dashed", color = "#009E73")+

geom_point(x = 1.23, y = 35, color = "maroon", size = 5)+ # Add the point at x = 7

geom_text(aes(label = 'LOQ=1.23'), x = 1.6, y = 36, vjust = -0.5, hjust = 1, color = "black", size = 4)+

theme_classic()+

theme(axis.text.x = element_text(angle = 0, vjust = 0.5, hjust = 1, size = 12),

axis.text.y = element_text(size = 12),

axis.title = element_text(size = 14),

legend.text = element_text(size = 12))

LOQ

**### statistical tests ###**

**# Welch t-test was performed on dPCR and ddPCR quantification results.**

# Allows for unequal variances

## T-test #####

## will need to do a separate test for each of the concentrations

# since the first two concentrations exceed 95% positive in either dPCR or ddPCR, we don't use them

subset_data3 <- subset(reformatted, expected_conc == 4.879513e+04 & Method %in% c("dPCR", "ddPCR"))

# Perform t-test

t_test_result <- t.test(Concentration ~ Method, data = subset_data3, mu=0, var.equal = FALSE)

# Print the result

print(t_test_result)

#subsetdata

subset_data4 <- subset(reformatted, expected_conc == 4.879513e+03 & Method %in% c("dPCR", "ddPCR"))

# Perform t-test

t_test_result <- t.test(Concentration ~ Method, data = subset_data4, mu=0, var.equal = FALSE)

# Print the result

print(t_test_result)

#subsetdata

subset_data5 <- subset(reformatted, expected_conc == 4.879513e+02 & Method %in% c("dPCR", "ddPCR"))

# Perform t-test

t_test_result <- t.test(Concentration ~ Method, data = subset_data5, mu=0, var.equal = FALSE)

# Print the result

print(t_test_result)

#subsetdata

subset_data6 <- subset(reformatted, expected_conc == 4.879513e+01 & Method %in% c("dPCR", "ddPCR"))

# Perform t-test

t_test_result <- t.test(Concentration ~ Method, data = subset_data6, mu=0, var.equal = FALSE)

# Print the result

print(t_test_result)

#subsetdata

subset_data7 <- subset(reformatted, expected_conc == 4.879513e+00 & Method %in% c("dPCR", "ddPCR"))

# Perform t-test

t_test_result <- t.test(Concentration ~ Method, data = subset_data7, mu=0, var.equal = FALSE)

# Print the result

print(t_test_result)

#subsetdata

subset_data8 <- subset(reformatted, expected_conc == 4.879513e-01 & Method %in% c("dPCR", "ddPCR"))

# Perform t-test

t_test_result <- t.test(Concentration ~ Method, data = subset_data8, mu=0, var.equal = FALSE)

# Print the result

print(t_test_result)

#subsetdata

subset_data9 <- subset(reformatted, expected_conc == 4.879513e-02 & Method %in% c("dPCR", "ddPCR"))

# Perform t-test

t_test_result <- t.test(Concentration ~ Method, data = subset_data9, mu=0, var.equal = FALSE)

# Print the result

print(t_test_result)

**### ANOVA ###**

### ANOVA to check if CV values are significantly different between the three methods

library(ggplot2)

library(dplyr)

library(cowplot)

data <- read.csv("02_real_DNA_methods-transformed.csv")

sperm_data <- filtered_data %>%

filter(DNA_type == "Sperm ")

sperm_data$individual <- factor(sperm_data$individual) ##To make the x-axis reflect the 10 individuals in your data, you need to specify individual as a factor variable.

### bloood

blood_data <- filtered_data %>%

filter(DNA_type == "Blood")

blood_data$individual <- factor(blood_data$individual) ##To make the x-axis reflect the 10 individuals in your data, you need to specify individual as a factor variable.

# Perform ANOVA on blood CV

anova_resultCVblood <- aov(CV_. ~ Method, data = blood_data)

# Check the ANOVA results

summary(anova_resultCVblood)

#Tukey’s HSD

TukeyHSD(anova_resultCVblood)

# Perform ANOVA on sperm CV

anova_resultCVbsperm <- aov(CV_. ~ Method, data = sperm_data)

# Check the ANOVA results

summary(anova_resultCVbsperm)

#Tukey’s HSD

TukeyHSD(anova_resultCVbsperm)
